# Supplementary material for: Effect of Different N:P Ratios on the Growth, Toxicity, and Toxin Profile of Gymnodinium catenatum (Dinophyceae) Strains from the Gulf of California
Source: Toxins (Basel). 2022 Jul 18;14(7):501. doi: 10.3390/toxins14070501 (PMC9321244; doi:10.3390/toxins14070501)
Supplement: Supplementary file 1 [file toxins-14-00501-s001.zip › toxins-1781043-supplementary.pdf]

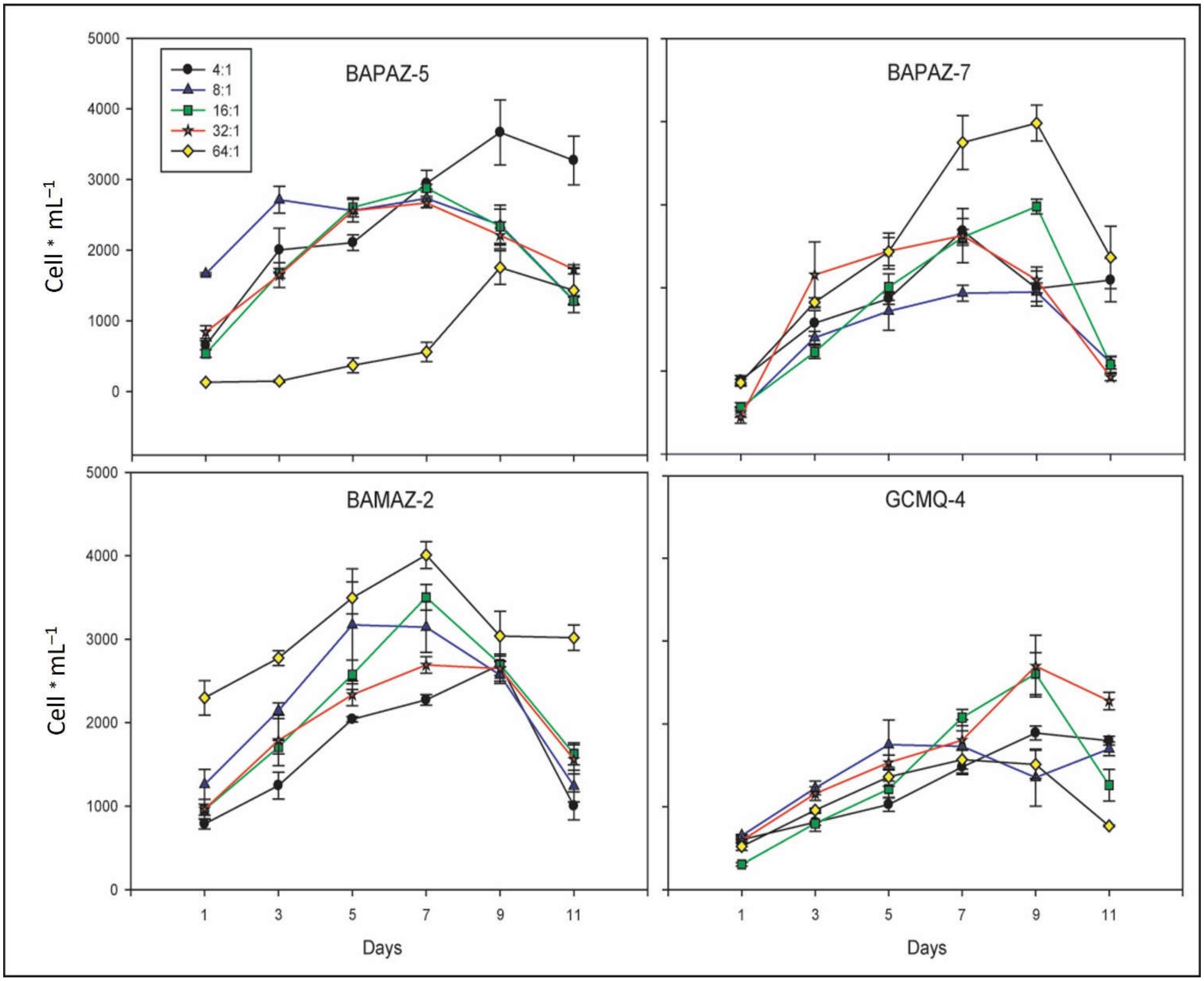

Supplementary Material Figure S1. Growth curves of *Gymnodinium catenatum* strains [i.e., GCMQ-4, BAMAZ-2, BAPAZ-5, and BAPAZ-7] grown with different N:P ratios in batch culture. The cell abundance shown are the average of triplicate culture.

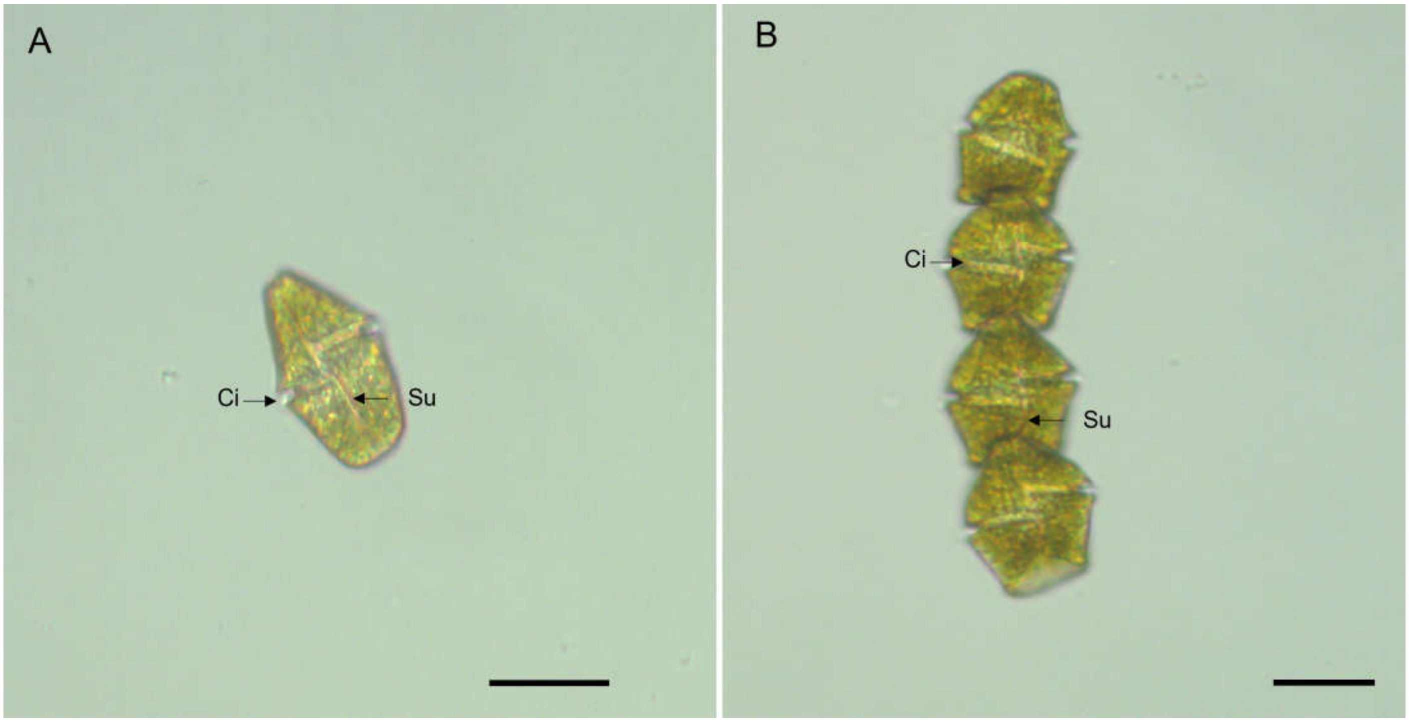

Supplementary Material Figure S2. Light micrographs of a Bahía de La Paz *Gymnodinium catenatum* strain. (A) Ventral view showing the cingulum and sulcus of single cell. (B) Ventral view of a four-celled chain showing cingulum and sulcus. Cingulum (Ci), Sulcus (Su). Scale bar: 20 μm. Micrographs: Leyberth José Fernández Herrera
